# An Improved Protocol for Comprehensive Etiological Characterization of Skin Warts and Determining Causative Human Papillomavirus Types in 128 Histologically Confirmed Common Warts

**DOI:** 10.3390/v14102266

**Published:** 2022-10-15

**Authors:** Lucijan Skubic, Lea Hošnjak, Vesna Breznik, Kristina Fujs Komloš, Boštjan Luzar, Mario Poljak

**Affiliations:** 1 Institute of Microbiology and Immunology, Faculty of Medicine, University of Ljubljana, Zaloška 4, 1000 Ljubljana, Slovenia; 2 Department of Dermatology and Venereal Diseases, Maribor University Medical Center, Ljubljanska 5, 2000 Maribor, Slovenia; 3 Institute of Pathology, Faculty of Medicine, University of Ljubljana, Korytkova 2, 1000 Ljubljana, Slovenia

**Keywords:** common warts, verrucae vulgares, etiological agent, human papillomavirus, *Alphapapillomavirus*, *Gammapapillomavirus*, viral load

## Abstract

Human papillomaviruses (HPVs) are etiologically associated with various benign and malignant neoplasms of cutaneous and mucosal epithelia. We describe an improved diagnostic protocol for comprehensive characterization of causative HPV types in common warts, in which broad-spectrum PCRs followed by Sanger sequencing, two previously described and seven newly developed type-specific quantitative real-time PCRs (qPCRs) coupled with the human beta-globin qPCR were used for: (i) diagnosis of HPV infection in warts; (ii) estimation of cellular viral loads of all HPV types detected; and (iii) determination of their etiological role in 128 histologically confirmed fresh-frozen common wart tissue samples. A total of 12 different causative HPV types were determined in 122/126 (96.8%) HPV-positive warts, with HPV27 being most prevalent (27.0%), followed by HPV57 (26.2%), HPV4 (15.1%), HPV2 (13.5%), and HPV65 (7.9%). The cellular viral loads of HPV4 and HPV65 were estimated for the first time in common warts and were significantly higher than the viral loads of HPV2, HPV27, and HPV57. In addition, we showed for the first time that HPV65 is etiologically associated with the development of common warts in significantly older patients than HPV27 and HPV57, whereas HPV4-induced warts were significantly smaller than warts caused by HPV2, HPV27, HPV57, and HPV65.

## 1. Introduction

Human papillomaviruses (HPVs) are a diverse group of small, non-enveloped DNA viruses that infect human cutaneous and mucosal stratified squamous epithelia, where they can cause the development of various benign and malignant neoplasms as well as persistent asymptomatic infections [1]. As of 15 September 2022, 223 HPV types have been officially recognized and are classified into five separate viral genera: *Alphapapillomavirus* (*Alpha*-PV), *Betapapillomavirus* (*Beta*-PV), *Gammapapillomavirus* (*Gamma*-PV), *Mupapillomavirus* (*Mu*-PV), and *Nupapillomavirus* (*Nu*-PV) [2]. Cutaneous HPVs from the *Alpha*-, *Gamma*-, *Mu*-, and *Nu*-PV genera are most commonly etiologically associated with the development of various types of skin warts, which are benign, self-limiting, and not life-threatening neoplasms, but they represent a significant public health problem due to their high incidence, often long-term persistence, and the various forms of discomfort and pain they can cause [3,4].

The most common type of skin warts are common warts (verrucae vulgares), which can occur as single or multiple lesions in people of all ages, but are more frequent in children and adolescents. Common warts typically appear as exophytic, hyperkeratotic, dome-shaped papules of varying sizes with a rough surface on exposed areas of the body, such as the fingers, palms, elbows, face, and knees, or as filiform variants mainly around the lips, eyelids, and nostrils [5,6]. Previous studies using various broad-spectrum polymerase chain reaction (PCR) methods followed by Sanger sequencing or hybridization with type-specific oligonucleotide probes have shown that cutaneous HPV types—HPV2, HPV27, and HPV57 from the *Alpha*-PV genus, HPV4 from the *Gamma*-PV genus, and HPV1 from the *Mu*-PV genus—predominate in common warts worldwide, independently of the immunocompromised status of patients [4,7,8,9,10,11,12,13,14,15], whereas HPV7 from the *Alpha*-PV genus is particularly associated with the so-called “butcher’s wart” variant of common warts, most often seen in meat handlers and fishmongers [5]. Meanwhile, HPV1 (*Mu*-PV genus), HPV4, and HPV65 (*Gamma*-PV genus) were reported to be more frequently present in common warts in Japan [16].

Although multiple HPV types can be detected in individual skin neoplasms, the HPV community supports the “one virus, one lesion” concept, according to which only a single HPV type is etiologically associated with the development of a specific lesion [11,17], whereas the other HPV types detected in a particular lesion are present due to contamination of the neoplasm’s surface [18] or as HPV bystanders with no clear active biological role [15,19]. To determine the etiology of common warts, it has been proposed to estimate type-specific cellular viral loads of HPV types detected in a given lesion using highly sensitive quantitative real-time PCR (qPCR) assays and based on estimated viral loads to identify which HPV type causes a particular skin wart (the so-called “causative HPV type”) and which HPV types are “non-causative” [20]. Thus, in most samples of common warts, wart-associated types HPV27 and HPV57 were shown to have high viral loads (up to 3.48 × 10^5^ and 3.02 × 10^5^ viral copies per cell, respectively), indicating the presence of productive viral infections and the etiological role of both HPV types in the development of skin warts, whereas viral loads of cutaneous HPV types from the *Beta*-PV genus were more than 100,000 times lower [20]. Subsequently, the cutoff value of one viral copy per cell was proposed to distinguish between causative versus non-causative (bystander) HPV types in individual common warts based on a clear bimodal distribution of estimated HPV viral loads in wart samples containing a single HPV infection (ranging from 5.0 × 10^−4^ to 7.1 × 10^6^ viral copies per cell) [15]. In our previous study, we detected the presence of HPV DNA in 176/185 (95.1%) fresh-frozen tissue samples of histologically confirmed common warts and then further analyzed 124 randomly selected HPV-positive wart samples. Using the initial diagnostic protocol and the proposed cutoff value, the most likely causative HPV type was determined in 99/124 (79.8%) samples (HPV57 being most frequent, followed by HPV27, HPV2, and HPV1), whereas in 25/124 (20.2%) warts the causative HPV type remained undetermined [15].

This study describes an improved diagnostic protocol for comprehensive detection and characterization of causative HPV types in fresh-frozen tissue samples of common warts, including a number of the most challenging warts containing multiple HPV types. We used broad-spectrum PCR assays followed by Sanger sequencing and two previously described and seven newly developed type-specific qPCR assays coupled with the human beta-globin (HBB) qPCR assay for: (i) diagnosis of HPV infection in wart tissue samples; (ii) estimation of cellular viral loads of all HPV types detected; and (iii) determination of their etiological role in the development of common warts. The improved diagnostic protocol described here made reliable determination of a causative HPV type possible in 122/128 (95.3%) histologically confirmed common warts. We then analyzed the potential differences in viral loads of the most frequent causative HPV types and the relationship between determined causative HPV types and selected demographic characteristics of patients and clinical features of common warts (sex and age of patients, and location and size of warts). To further test the robustness of the improved diagnostic protocol, we additionally used this protocol to analyze all common wart samples without detectable HPV DNA and all HPV-positive warts without a reliably determined causative HPV type from our previous study [15].

## 2. Materials and Methods

### 2.1. Tissue Samples

The main study population was composed of 128 fresh-frozen tissue samples of histologically confirmed common warts collected from 90 immunocompetent patients treated in the Department of Dermatology and Venereal Diseases, Maribor University Medical Center, Slovenia. Among them, 52 samples obtained from 38 patients were collected between February 2014 and April 2016 as part of our previous study [15]; after randomization, none of these 52 samples was included in the etiological characterization of common warts in our previous study. In addition, 76 samples obtained from 52 patients were collected de novo between March 2020 and May 2022 for the purpose of this study. The common warts were located on the hands, arms, legs, including dorsal sides of the feet, and on the head of the patients. Plantar warts or periungual warts were not included in our study.

Using the improved diagnostic protocol, we separately analyzed an additional 34 samples from our previous study [15]: (i) nine samples in which HPV DNA was not detected using our initial diagnostic protocol; and (ii) 25 otherwise HPV-positive samples in which the causative HPV types could not be successfully determined using the initial protocol for etiological characterization of common warts.

**Figure 1:**
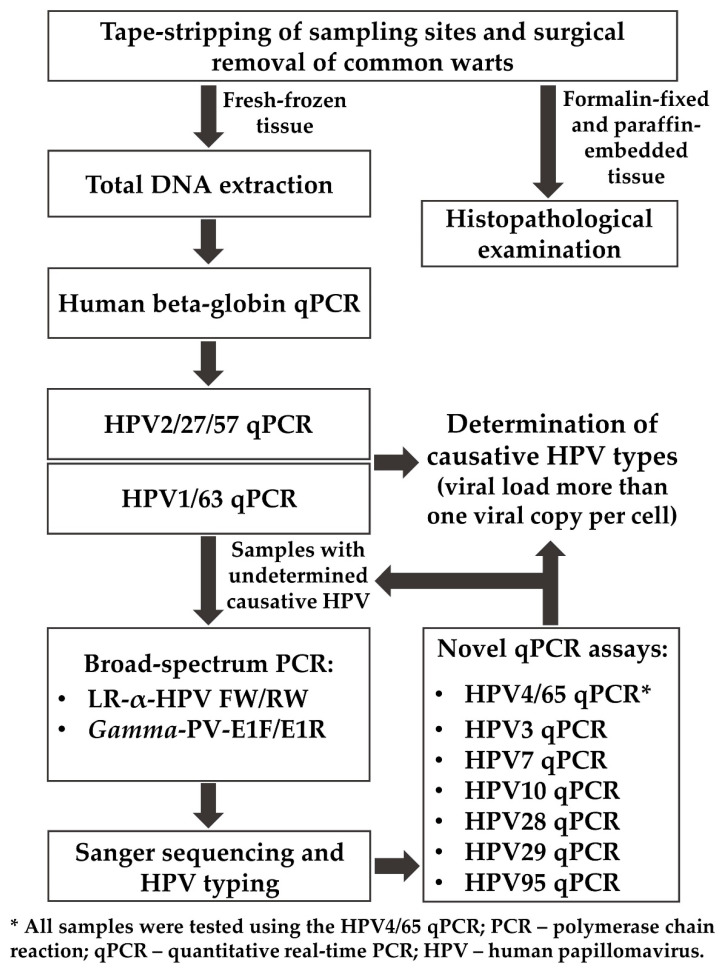
Protocol for the comprehensive characterization of causative human papillomavirus (HPV) types in fresh-frozen tissue samples of histologically confirmed common warts.

Prior to surgical removal, each wart was examined with a digital dermatoscope, photographed, and tape-stripped to reduce the probability of contamination of the skin surface with HPVs that did not establish the infection (Figure 1) [18]. Samples were collected by 3–4 mm punch biopsies after local anesthesia and divided into two parts; one part of each sample was immediately stored at −80 °C in a liquid nitrogen dewar for DNA extraction and downstream PCR analyses, and the second part was fixed with 10% neutral buffered formalin and subsequently embedded into paraffin for routine histological hematoxylin and eosin staining. In cases in which a single patient had multiple warts, only warts that were more than 1 cm apart were sampled to reduce potential cross-contamination between different samples [15,21].

The study was conducted in compliance with the Declaration of Helsinki and was approved by the Ethics Committee of the Ministry of Health of the Republic of Slovenia (consent references 63/10/13 and 0120-87/2020/3). All patients signed an informed consent form before participating in the study.

### 2.2. Total DNA Extraction and Quality Verification

Total DNA from fresh-frozen tissue samples of common warts was extracted using the QIAamp DNA Mini Kit (Qiagen, Hilden, Germany) according to the manufacturer’s protocol for tissue DNA extraction (Figure 1). Subsequently, the extracted DNA was quantified with a Qubit 4 Fluorometer (Thermo Scientific, Waltham, MA, USA) using the Qubit dsDNA HS Assay Kit (Thermo Scientific) as instructed by the manufacturer and stored at −20 °C until further use.

The integrity of the extracted DNA and absence of PCR inhibitors were verified in all samples by qPCR amplification of a 150 base-pair (bp) fragment of the single-copy HBB gene using the specific qPCR primer-probe set (Figure 1, Table 1) [22]. The HBB qPCR assay was performed using the LightCycler 480 Probes Master kit (Roche Diagnostics, Mannheim, Germany) in a 20 μL reaction mixture, containing 5 µL of sample DNA (up to 100 ng), 10 µL of 2× LightCycler 480 Probes Master, 0.4 μM of each of the primers, 0.2 μM of the probe, and nuclease-free water. The qPCR reaction targeting the HBB gene was conducted on a LightCycler 480 Instrument II (Roche Diagnostics) under the following conditions: initial DNA denaturation for 10 min at 95 °C (temperature transition rate of 4.4 °C/s), followed by 45 amplification cycles of 10 s at 95 °C (4.4 °C/s), 30 s at 60 °C (2.2 °C/s), and 1 s at 72 °C (4.4 °C/s). Acquisition of the fluorescence signal (510 nm) was performed in a single mode at the end of the elongation step of each amplification cycle. The final step consisted of cooling the reaction mixture to 40 °C with a 30 s hold (2.2 °C/s). The qPCR results were analyzed using LightCycler 480 Software v1.5.1.62 (Roche Diagnostics).

The analytical sensitivity of the HBB qPCR assay was determined as described previously [23,25] by testing triplicates of 10-fold serially diluted standards of human genomic DNA (Roche Diagnostics), corresponding to an input of 1 × 10^4^ to 1 × 10^−1^ HBB gene copies per reaction. The detection limit of the HBB qPCR assay was established at 10 HBB gene copies per reaction. The calculated coefficient of determination of the HBB qPCR standard curve was 0.999, and the amplification efficiency was estimated at 97.2%.

### 2.3. HPV DNA Detection and Typing

To determine the presence of the most common cutaneous HPV types, each sample was first tested using the HPV2/27/57 multiplex type-specific qPCR assay (Figure 1, Table 1), allowing sensitive detection and differentiation of HPV2, HPV27, and HPV57, as described previously [23]. Real-time monitoring of the fluorescent signal was performed on 510, 580, and 660 nm channels, respectively. Subsequently, the determination of HPV1 and HPV63 infection in each sample was performed using the HPV1/63 duplex type-specific qPCR assay (Figure 1, Table 1), as described previously [24].

The presence of a wide spectrum of HPV types from the *Alpha*- and *Gamma*-PV genera was tested in all HPV qPCR-negative samples and in samples with minute HPV viral loads using two sets of broad-spectrum primers: (i) LR-α-HPV FW/RW, targeting an approximately 190-bp fragment of the E1 genomic region of at least 23 different low-risk HPV types from the *Alpha*-PV genus (HPV2, −3, −6, −7, −10, −11, −13, −27, −28, −29, −32, −40, −42, −43, −44, −55, −57, −74, −77, −91, −94, −117, and −125); and (ii) *Gamma*-PV-E1F/E1R, targeting an approximately 500-bp fragment of the E1 genomic region of at least eight different HPV types from the *Gamma*-PV genus (HPV4, −48, −50, −60, −65, −88, −101, and −103), as described previously (Figure 1) [26,27,28]. The concentration of each of the *Gamma*-PV-E1F/E1R primers in the reaction mixture was 0.75 μM. The PCR products obtained were analyzed with 1% agarose gel electrophoresis and purified using the QIAquick PCR Purification Kit (Qiagen) following the manufacturer’s instructions. Sanger sequencing of the purified HPV-positive PCR products on an automated ABI3500 Genetic Analyzer sequencing instrument (Applied Biosystems, Life Technologies, Carlsbad, CA, USA) and HPV type determination were performed on both DNA strands, as described previously [29,30]. The size of the sequences generated by direct sequencing of LR-α-HPV FW/RW and *Gamma*-PV-E1F/E1R PCR products and used for HPV type assignment was approximately 130 and 440 bp, respectively.

According to the results of sequencing-based HPV typing, HPV3-, HPV4-, HPV7-, HPV10-, HPV28-, HPV29-, HPV65-, and HPV95-specific qPCR primer-probe sets (Table 1) were designed and evaluated using Primer3 v0.4.0 (https://bioinfo.ut.ee/primer3-0.4.0/, accessed on 20 February 2022) [31,32] and Net Primer (http://www.premierbiosoft.com/netprimer/, accessed on 20 February 2022) web-based applications, respectively. The nucleotide sequences of reference HPV genomes used as templates for the design of type-specific primers and double-quenched probes were obtained from the Papillomavirus Episteme [33] and GenBank databases (https://www.ncbi.nlm.nih.gov/genbank/, accessed on 20 February 2022), under the accession numbers X74462 (HPV3), X70827 (HPV4), X74463 (HPV7), X74465 (HPV10), U31783 (HPV28), U31784 (HPV29), X70829 (HPV65), and AJ620210 (HPV95). To avoid potential cross-reactivity with non-targeted sequences, the specificity of the designed primers and probes was additionally verified using the NCBI Nucleotide BLAST web-based service (https://blast.ncbi.nlm.nih.gov/Blast.cgi, accessed on 20 February 2022). Due to the differences in genomic nucleotide sequences and their chemical and thermodynamic properties, it was more convenient to develop type-specific qPCR assays targeting different genomic regions of individual HPV types to achieve efficient HPV DNA PCR amplification.

To test the presence of identified HPV types by Sanger sequencing, all samples were subjected to qPCR amplification of 122 and 130 bp fragments of the HPV4 and HPV65 L2 genomic regions, respectively, using the newly developed HPV4- and HPV65-specific qPCR primer-probe sets (Figure 1). The HPV4/65 duplex type-specific qPCR assay was conducted using the LightCycler 480 Probes Master kit (Roche Diagnostics) in a 20 μL reaction mixture, containing 5 µL of sample DNA (up to 100 ng), 10 µL of 2× LightCycler 480 Probes Master, 0.5 μM of each of the primers, 0.1 μM of each of the probes, and nuclease-free water. The HPV4/65 duplex qPCR protocol was performed on a LightCycler 480 Instrument II (Roche Diagnostics) under the same conditions as the HBB qPCR assay described above, and the amplifications were monitored by measuring the fluorescence signal at the end of each elongation step on 510 and 580 nm channels, respectively. Meanwhile, the newly developed HPV3-, HPV7-, HPV10-, HPV28-, HPV29-, and HPV95-specific qPCR primer-probe sets were used in singleplex type-specific qPCR assays conducted in the same way as the HBB qPCR assay, testing only individual samples in which specific HPV types were identified by Sanger sequencing (Figure 1).

Testing triplicates of 10-fold serially diluted reference plasmids, containing the target HPV3, HPV4, HPV7, HPV28, HPV29, HPV65, and HPV95 genomic regions, respectively, at concentrations of 1 × 10^9^ to 1 × 10^−1^ copies of viral DNA per reaction, and reference plasmids, containing the target HPV10 genomic region at concentrations of 1 × 10^8^ to 1 × 10^−1^ copies of viral DNA per reaction, showed that all the type-specific qPCR assays mentioned had a sensitivity of at least 10 viral genome equivalents per reaction. The calculated coefficient of determination of all qPCR standard curves was 0.999, and the amplification efficiencies were estimated at 94.2% for HPV3, 95.0% for HPV4, 96.7% for HPV7, 97.0% for HPV10, 92.3% for HPV28, 96.0% for HPV29, 97.2% for HPV65, and 95.7% for HPV95.

A reaction mixture with no DNA template was used in all PCR runs as a reference control for potential amplicon carryover contamination.

Reference plasmids containing partial or complete HPV genomic DNA were kindly provided by the International HPV Reference Center (Karolinska Institute, Stockholm, Sweden)–E.-M. de Villiers, German Cancer Research Center, Heidelberg, Germany (HPV1, HPV3, HPV4, HPV7, HPV63, and HPV95), M. Favre, Pasteur Institute, Paris, France (HPV10, HPV28, and HPV29), and T. Matsukura, National Institute of Health, Tokyo, Japan (HPV65).

### 2.4. Viral Load Calculation

To determine the cellular viral load of HPVs in common warts, HPV type-specific qPCR assays were used in combination with the qPCR assay targeting the HBB gene described above. HBB gene and HPV concentrations (copies/μL) were calculated from the quantification cycle (Cq) value of sample DNA using standard curves generated by plotting Cq values against known concentrations of input human genomic DNA (Roche Diagnostics) or HPV reference plasmids. In the calculations, it was assumed that 3.3 pg of human genomic DNA contained one copy of the HBB gene [25]. Ratios between the number of viral copies and human diploid cells were calculated as follows: (viral copies/(HBB gene copies/2)). Any HPV type with a viral load of more than one viral copy per cell in a specific wart sample was considered a causative HPV type of that particular wart (Figure 1) [15].

### 2.5. Statistical Analyses

Data management, analysis, and graphic presentation were performed with RStudio v1.2.5033 software (R version 3.6.3; RStudio, Inc., Boston, MA, USA). The descriptive statistics are presented as number and percentage for categorical variables. For numerical variables, the minimum, maximum, arithmetic mean, standard deviation, and median were calculated. The Mann–Whitney *U* test was used to evaluate differences in age distribution between patients of both sexes and to compare estimated viral loads between two different groups of causative HPVs. The Kruskal–Wallis test was used to compare patients’ age, wart size, and estimated viral loads between different causative HPV types. The Fisher’s exact test was used to determine associations between patients’ sex and the number of common warts caused by HPV27. Results were considered statistically significant at *p*-value ≤0.05.

## 3. Results

### 3.1. Characteristics of Patients and Common Warts

**Figure 2:**
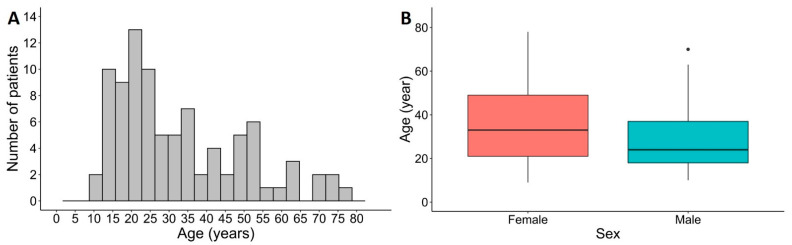
Demographic characteristics of immunocompetent patients with common warts included in the study. (**A**) Age distribution of patients (*n* = 90). (**B**) Age distribution of patients by sex. The median age of females (*n* = 47) was 33 years, and the median age of males (*n* = 43) was 24 years.

The main study population consisted of 90 immunocompetent patients (median age: 27.5 years; age range: 9–78 years), including 47/90 females (52.2%) and 43/90 males (47.8%; Figure 2A and Table S1). The median age of females was 33 years, while the median age of males was 24 years (*p* = 0.094, Mann–Whitney *U* test; Figure 2B).

At study entry, a single common wart was obtained from 57/90 patients (63.3%), two warts from 28/90 patients (31.1%), and three different warts from 5/90 patients (5.6%; Table S1). The median duration of the presence of common warts was 12 months, ranging from 1 to 120 months. Of the 128 common warts sampled, 96/128 (75.0%) warts were located on the hands including wrists, 13/128 (10.1%) on the arms, 12/128 (9.4%) on the legs including dorsal sides of feet, and 7/128 (5.5%) on the head of the patients. The median size of the common warts collected was 5 mm in diameter, ranging from 2 to 20 mm.

### 3.2. HPV DNA Detection and Typing

The 150 bp fragment of the HBB gene, serving as an internal control, was successfully amplified from all 128 fresh-frozen tissue samples of histologically confirmed common warts included in the study, indicating adequate quality of the sample DNA for subsequent HPV testing.

Using type-specific qPCR assays and broad-spectrum PCR assays followed by Sanger sequencing analysis, HPV DNA was detected in 126/128 (98.4%) samples of histologically confirmed common warts, with single and multiple HPV infections detected in 88/126 (69.8%) and 38/126 (30.2%) samples, respectively (Table 2, Table S1). As shown in Table 2, a total of 13 different HPV types from three viral genera–*Alpha*-, *Gamma*-, and *Mu*-PV, and six viral species–*Alpha*-PV species 2, 4, 8 (*Alpha*-2, 4, 8), *Gamma*-PV species 1 (*Gamma*-1), and *Mu*-PV species 1, 2 (*Mu*-1, 2) were identified. HPV27 (40/126; 31.7%) and HPV57 (38/126; 30.2%) were the most frequently detected HPV types in common warts, followed by HPV4 (23/126; 18.3%), HPV2 (19/126; 15.1%), HPV65 (19/126; 15.1%), HPV1 (15/126; 11.9%), and HPV10 (5/126; 4.0%). HPV63 and HPV28 were detected in three and two different common wart samples, respectively, and HPV3, HPV7, HPV29, and HPV95 were each identified in a single wart sample.

### 3.3. Causative HPV Type Determination

Based on the estimation of cellular viral loads by the type-specific qPCR assays and applying the cutoff value for determination of the causative HPV type of one viral copy per cell [15], a causative HPV type was reliably identified in 122/126 (96.8%) HPV-positive common warts, including several samples with multiple HPVs (Table S1). As shown in Table 3, the most frequent causative HPV type in common warts was HPV27 with a prevalence rate of 34/126 (27.0%), followed by HPV57 (33/126; 26.2%), HPV4 (19/126; 15.1%), HPV2 (17/126; 13.5%), and HPV65 (10/126; 7.9%). HPV10 and HPV28 were etiologically associated with the development of two common warts, and HPV1, HPV3, HPV7, HPV29, and HPV95 were each determined as etiological agents in a single wart. Among the five most frequent causative HPV types, HPV2 had the highest proportion (17/19; 89.5%) of common wart samples in which it was identified as the etiological agent according to the number of samples in which it was detected (i.e., the caused-detected proportion), whereas HPV65 had the lowest caused-detected proportion (10/19; 52.6%). Although HPV63 was detected in three common warts, it could not be identified as the causative HPV in any of the warts due to its minute viral load.

In 33 patients from whom multiple common wart samples were collected, 29/33 (87.9%) had a complete concordance of the determined causative HPVs. The exceptions were a 15-year-old male patient (Patient no. 35; Table S1) whose common warts on the right hand were caused by HPV4 and HPV65, respectively, and a 22-year-old male patient (Patient no. 14; Table S1) and a 25-year-old female patient (Patient no. 52; Table S1) in whom the causative HPV types (HPV2 and HPV4; HPV4 and HPV57) of warts on the hands were classified into two different viral genera: *Alpha*- and *Gamma*-PV, respectively. In addition, in a 41-year-old male patient (Patient no. 89; Table S1), a common wart on the right hand was caused by HPV10, whereas HPV1 and HPV10 with minute viral loads were detected in an adjacent wart.

Overall, in our main study population, out of 128 histologically confirmed common warts, the causative HPV types could not be reliably assigned in only four wart samples containing all HPV types detected with estimated viral loads of less than one viral copy per cell (Samples nos. 29, 78, 125, and 127; Table S1) and in two wart samples in which no HPV DNA was detected (Samples nos. 107 and 110; Table S1).

### 3.4. Viral Load of Causative HPV Types

**Figure 3:**
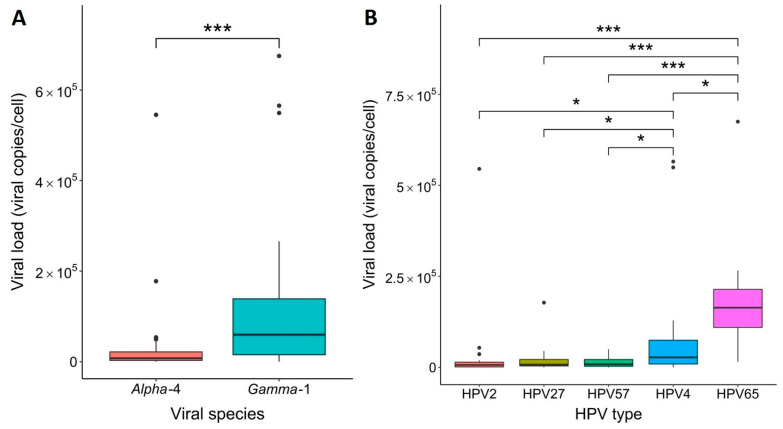
Estimated cellular viral loads of the most frequent causative human papillomaviruses (HPVs) in common warts. (**A**) Comparison of viral loads of causative HPVs from the *Alpha*-4 (median viral load: 7.73 × 10^3^ copies/cell) and *Gamma*-1 species (median viral load: 5.94 × 10^4^ copies/cell). Statistical comparison was performed with the Mann–Whitney *U* test (***, *p* < 0.0001). (**B**) Comparison of viral loads among the five most frequent causative HPV types. The viral load of HPV4 (median viral load: 2.75 × 10^4^ copies/cell) was significantly higher than the viral loads of HPV2 (median viral load: 6.73 × 10^3^ copies/cell; *, *p* = 0.030), HPV27 (median viral load: 7.73 × 10^3^ copies/cell; *, *p* = 0.026), and HPV57 (median viral load: 8.65 × 10^3^ copies/cell; *, *p* = 0.017). The viral load of HPV65 (median viral load: 1.64 × 10^5^ copies/cell) was significantly higher than the viral loads of HPV2 (***, *p* < 0.001), HPV4 (*, *p* = 0.014), HPV27 (***, *p* < 0.0001), and HPV57 (***, *p* < 0.0001). Statistical comparison was performed with the Kruskal–Wallis test.

The median cellular viral load of HPV2, HPV27, and HPV57 from the *Alpha*-4 species in the caused common warts was estimated to be 7.73 × 10^3^ copies/cell, and was significantly lower than the estimated viral loads of HPV4, HPV65, and HPV95 from the *Gamma*-1 species (median viral load: 5.94 × 10^4^ copies/cell; *p* < 0.0001, Mann–Whitney *U* test; Figure 3A). As shown in Figure 3B and Table 4, further detailed analysis using the Kruskal–Wallis test confirmed that the estimated viral load of HPV4 (median viral load: 2.75 × 10^4^ copies/cell) was significantly higher than the viral load of HPV2 (median viral load: 6.73 × 10^3^ copies/cell; *p* = 0.030), HPV27 (median viral load: 7.73 × 10^3^ copies/cell; *p* = 0.026), and HPV57 (median viral load: 8.65 × 10^3^ copies/cell; *p* = 0.017). Meanwhile, the estimated viral load of HPV65 (median viral load: 1.64 × 10^5^ copies/cell) was significantly higher than the viral loads of HPV2 (*p* < 0.001), HPV4 (*p* = 0.014), HPV27 (*p* < 0.0001), and HPV57 (*p* < 0.0001). The mean of HPV10 and HPV28 viral loads in the caused common warts was estimated to be 3.54 × 10^3^ and 4.17 × 10^5^, respectively, and the viral loads of HPV1, HPV3, HPV7, HPV29, and HPV95 were estimated to be 2.53 × 10^4^, 2.17 × 10^4^, 1.75 × 10^1^, 1.02 × 10^4^, and 5.87 × 10^2^ copies/cell, respectively (Table S1).

### 3.5. Relationship between Causative HPV Types and Patient Characteristics

The proportion of HPV2 as the etiological agent of common warts was the same in patients of both sexes, whereas HPV4, HPV57, and HPV65 were identified as the causative HPV types in more female than male patients (Table 3). In contrast, HPV27 caused common warts in 16/43 (37.2%) males and 9/47 (19.1%) females and was the predominant etiological agent of warts in male patients. However, the Fisher’s exact test did not confirm that there was a statistically significant association between patients’ sex and the number of common warts caused by HPV27 (*p* = 0.064, odds ratio = 2.476, 95% confidence interval: 0.877–7.387).

**Figure 4:**
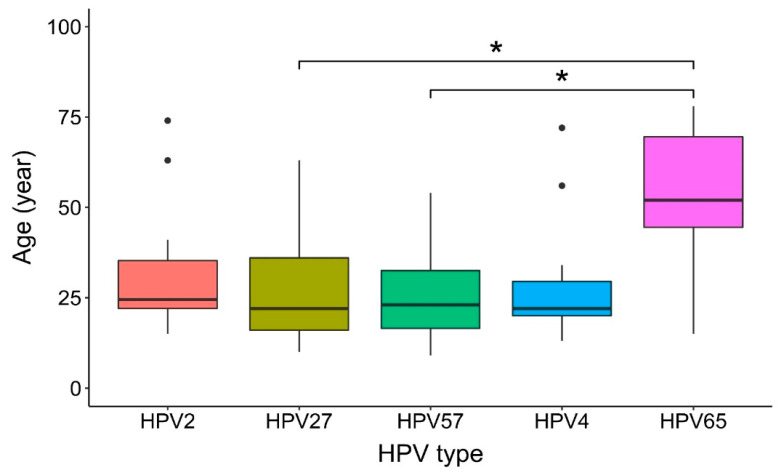
Comparison of patients’ age among the five most frequent causative human papillomavirus (HPV) types in common warts. HPV65 was more frequently etiologically associated with the development of common warts in significantly older patients (median age: 52 years) than HPV27 (median age: 22 years; *, *p* = 0.047) and HPV57 (median age: 23 years; *, *p* = 0.047). Statistical comparison was performed with the Kruskal–Wallis test and considered that three patients with multiple samples of common warts had different causative HPV types in different warts.

Comparison of patients’ age among the most frequent causative HPV types using the Kruskal–Wallis test showed that HPV65 was more frequently etiologically associated with the development of common warts in significantly older patients (median age: 52 years) than HPV27 (median age: 22 years; *p* = 0.047) and HPV57 (median age: 23 years; *p* = 0.047), whereas there was no significant difference with the age of patients with HPV2 (median age: 24.5 years; *p* = 0.157) and HPV4-induced warts (median age: 22 years; *p* = 0.157; Figure 4 and Table 3).

In the statistical analysis, it was considered that three patients with multiple samples of common warts had different causative HPV types in different warts (Patients nos. 14, 35, and 52; Table S1).

### 3.6. Relationship between Causative HPV Types and Clinical Features of Common Warts

The most frequent causative HPV type in common warts on the hands was HPV27 with a prevalence rate of 25/96 (26.0%), followed by HPV57 (19/96; 19.8%), HPV4 (19/96; 19.8%), HPV2 (13/96; 13.5%), HPV65 (9/96; 9.4%), HPV28 (2/96; 2.1%), HPV1 (1/96; 1.0%), HPV7 (1/96; 1.0%), HPV10 (1/96; 1.0%), HPV29 (1/96; 1.0%), and HPV95 (1/96; 1.0%; Table S1). Whereas HPV3, HPV10, and HPV65 were each etiologically associated with the development of a single wart on the arms (3/13; 23.1%), HPV27 was also the most prevalent etiological agent in common warts in this anatomical region (4/13; 30.8%), followed by HPV2 (3/13; 23.1%) and HPV57 (3/13; 23.1%). Furthermore, HPV57 was the most frequent causative HPV type in common warts on the legs (5/12; 41.7%), followed by HPV27 (4/12; 33.3%) and HPV2 (1/12; 8.3%). All seven common warts obtained from the head area were caused by HPV57 (6/7; 85.7%) or HPV27 (1/7; 14.3%).

**Figure 5:**
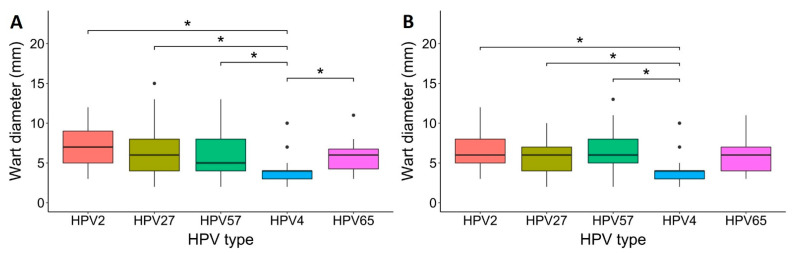
The size of common warts caused by the five most frequent causative human papillomavirus (HPV) types. (**A**) Comparison of the size of common warts at different anatomical sites among the causative HPV types. HPV4 caused significantly smaller common warts (median diameter: 4 mm) than HPV2 (median diameter: 7 mm; *, *p* = 0.021), HPV27 (median diameter: 6 mm; *, *p* = 0.030), HPV57 (median diameter: 5 mm; *, *p* = 0.041), and HPV65 (median diameter: 6 mm; *, *p* = 0.041). (**B**) Comparison of the size of common warts on the hands among causative HPV types. HPV4 caused significantly smaller common warts (median diameter: 4 mm) than HPV2 (median diameter: 6 mm; *, *p* = 0.017), HPV27 (median diameter: 6 mm; *, *p* = 0.056), and HPV57 (median diameter: 6 mm; *, *p* = 0.017). Both statistical comparisons were performed with the Kruskal–Wallis test.

Comparison of the size of common warts at various anatomical sites among different causative HPV types using the Kruskal–Wallis test revealed that HPV4 was etiologically associated with the development of significantly smaller common warts (median diameter: 4 mm) than HPV2 (median diameter: 7 mm; *p* = 0.021), HPV27 (median diameter: 6 mm; *p* = 0.030), HPV57 (median diameter: 5 mm; *p* = 0.041), and HPV65 (median diameter: 6 mm; *p* = 0.041; Figure 5A). In an additional statistical analysis that included only common warts obtained from the hands, the Kruskal–Wallis test confirmed that HPV4 was etiologically associated with the development of significantly smaller common warts on the hands (median diameter: 4 mm) than HPV2 (median diameter: 6 mm; *p* = 0.017), HPV27 (median diameter: 6 mm; *p* = 0.056), and HPV57 (median diameter: 6 mm; *p* = 0.017), whereas the size of warts caused by HPV65 did not differ significantly (median diameter: 6 mm; *p* = 0.070; Figure 5B).

### 3.7. Robustness of the Improved Diagnostic Protocol for Comprehensive Etiological Characterization of Common Warts

As summarized in Table S2, using the improved protocol described for comprehensive etiological characterization of common warts, all nine common warts that tested HPV DNA-negative using the initial diagnostic protocol in our previous study [15] were now HPV-positive. A causative HPV type was reliably determined in 7/9 (77.8%) common warts, with HPV4 (4/9 samples; 44.4%) and HPV65 (3/9; 33.3%) being the only etiological agents identified (Table S2).

As shown in Table S3, using the improved protocol described, a causative HPV type was identified in 18/25 (72.0%) wart samples in which the etiological agent could not be reliably determined in our previous study [15]. The most frequent causative HPV type in these warts was HPV4, which was identified in 9/25 (36.0%) samples, followed by HPV65 (4/25; 16.0%), HPV7 (3/25; 12.0%), HPV10 (1/25; 4.0%), and HPV29 (1/25; 4.0%; Table S3).

## 4. Discussion

Most previous research on the presence of HPVs in common warts has focused on studying the prevalence and diversity of HPV types, often evaluating the etiological association between HPV and warts based only on high HPV prevalence without reliably confirming the presumed etiological links [4,7,9,10,11,12,13,14]. This study describes an improved diagnostic protocol for comprehensive detection and characterization of causative HPV types in more than 95% of common warts based on the estimation of HPV type-specific cellular viral loads using seven newly developed type-specific qPCR assays targeting eight different HPV types. The improved diagnostic protocol also allowed reliable determination of the causative HPV types in the great majority of the most challenging common warts; for example, those containing multiple HPV types and those that are HPV DNA-negative or HPV-positive but without a reliably determined causative HPV type in our previous study [15]. We also present for the first time the differences in viral loads of the most frequent causative HPV types and the relationship between determined causative HPV types and selected demographic characteristics of patients and clinical features of common warts.

Using several different molecular approaches, including broad-spectrum PCR assays followed by Sanger sequencing and highly sensitive qPCR assays for HPV detection and typing [23,24,26,27,30], we tested 128 fresh-frozen biopsies of common warts that had been previously confirmed histologically. Although cutaneous neoplasms can often be misdiagnosed clinically and histopathological examination is considered the diagnostic gold standard [34,35,36], several previous studies assessed the presence of HPV in common warts that were diagnosed only clinically [7,13,14,16] and/or used for HPV detection skin swabs of the wart’s surface instead of the wart’s tissue [4,11,12,37]. Skin biopsies yield important information on HPV DNA localization throughout the entire thickness of the neoplasms and thus, coupled with type-specific viral load determination, allow reliable confirmation of causative HPV types [20,35]. In contrast, non-invasive skin swabs only provide information on the presence of HPVs in the superficial layers of the warts, where HPVs may have been present not only as a result of productive infection (causing development of warts) but also as colonization or contamination of the skin surface [15,18]. To reduce the probability of contamination of the skin surface with HPVs that have not established a productive infection, in this study we tape-stripped sampling sites prior to surgical removal of common warts using the established procedure [18].

The overall prevalence of HPVs detected in fresh-frozen tissue samples of histologically confirmed common warts in our main study population was 126/128 (98.4%), which is more or less consistent with previous studies in which HPV positivity ranged from 82.4 to 100% [7,8,10,11,12,13,15,16]. Multiple HPV types were detected in 30.2% of common wart samples, which is significantly higher compared to similar studies, in which 1.8 to 12.3% of common warts had multiple HPV infections [3,7,10,14]. This is most probably due to the use of highly sensitive type-specific qPCR assays for HPV typing in this study [23,24]. In previous research, the most frequently detected HPV types in common warts worldwide were HPV2 (in 10.4–34.7% of samples), HPV27 (20.8–31.1%), and HPV57 (12.4–35.2%) from the *Alpha*-PV genus, HPV4 (1.8–19.8%) from the *Gamma*-PV genus, and HPV1 (3.3–31.3%) from the *Mu*-PV genus [7,10,11,12]. Similarly, the most prevalent HPV type in this study was HPV27 (in 31.7% of HPV-positive common warts), followed by HPV57 (30.2%), HPV4 (18.3%), HPV2 (15.1%), HPV65 (15.1%), and HPV1 (11.9%). Interestingly, HPV65 from the *Gamma*-PV genus has been previously reported to be more prevalent in common warts on the hands in Japan (in 14.1% of samples) [16] than in other populations (up to 3.8% of samples) [11,12,38]. Other HPV types detected in common warts in this study were HPV3, HPV7, HPV10, HPV28, and HPV29 from the *Alpha*-PV genus, HPV63 from the *Mu*-PV genus, and HPV95 from the *Gamma*-PV genus, which were also rarely detected previously [8,11,12,13,16].

To identify causative HPV types in common warts based on the estimation of cellular viral loads of all 13 HPVs detected, we used existing type-specific qPCR assays targeting five HPV types in combination with seven newly developed type-specific qPCR assays targeting eight different HPV types. Consistent with the “one virus, one lesion” concept, the single causative HPV type was reliably identified in 96.8% of HPV-positive common warts in this study, whereas the causative HPV type remained undetermined in four samples. The proportion of common warts with an identified etiological agent was thus much higher than in our previous study, in which etiological agents were successfully assigned in 79.8% of the warts analyzed [15]. In agreement with previous research [11,15], almost nine out of 10 patients from whom multiple common wart samples were collected had a complete concordance of identified causative HPVs identified in various warts, further confirming that a single HPV type is typically involved in the development of multiple warts in an individual patient.

Based on the cutoff value for the determination of the causative HPV type of one viral copy per cell [15], this study further confirmed that HPV2, HPV27, and HPV57 from the *Alpha*-4 species are the most frequent etiological agents in common warts [15,20] and has further shown that HPV4 and HPV65 from the *Gamma*-1 species are also important causative HPV types with high prevalence. The least common causative HPV types in this study were HPV3, HPV10, HPV28, and HPV29 from the *Alpha*-2 species, which were previously predominantly described in flat warts (verrucae planae) [5,12], HPV7 from the *Alpha*-8 species, which is most often seen in the “butcher’s wart” variant of common warts in individuals whose hands are chronically exposed to moisture and cold [5,39], and HPV95, which is a rare cutaneous HPV type from the *Gamma*-1 species [40]. Previous studies have shown that HPV1 from the *Mu*-1 species is frequently identified in clinically normal skin and in skin warts of patients in primary healthcare, especially in children under 12 years of age, with a distinct clinical profile because HPV1-induced warts resolve relatively quickly without treatment (i.e., in less than 6 months) [7,11,37]. In this study, HPV1 was present in 15 samples of common warts with predominantly multiple HPV infections and was subsequently identified as the etiological agent in a single wart only, possibly because the median age of patients in the cohort studied was 27.5 years (with a range of 9 to 78 years) and only two patients were younger than 12 years, most likely resulting in an underrepresentation of the prevalence of warts caused by HPV1.

In this study, the causative HPV type was not determined in only six of 128 samples of common warts in which either HPV DNA was not detected or the estimated viral load of the HPV types present was less than one viral copy per cell. In our previous study, using the initial diagnostic protocol, HPV DNA was not detected in 9/185 (4.9%) common warts, and no etiological agent was found in 25/124 (20.2%) randomly analyzed HPV-positive common warts, which was primarily a consequence of the suboptimal methodological approach because the presence of HPVs from the *Gamma*-PV genus was not tested and the cellular viral load of only six detected HPV types was estimated (i.e., HPV1, HPV2, HPV27, HPV57, HPV63, and HPV204) [15]. Using the improved diagnostic protocol described here, which allows detection of *Gamma*-HPVs and estimation of the cellular viral load of 13 HPV types, we determined the etiological agents in 7/9 (77.8%) previously “HPV-negative” common wart samples and in 18/25 (72.0%) HPV-positive warts without previously assigned causative HPV types [15], with HPV4 and HPV65 being the most frequent etiological agents, followed by HPV7, HPV10, and HPV29. In addition to the methodological reasons described above, it was previously shown that skin warts that existed longer were less likely to contain HPV DNA [4], which may be the reason that the causative HPV type could not be identified in some of the warts included in this study, particularly those with long wart duration (i.e., 60 and 120 months). Nevertheless, there is a possibility that other HPVs not targeted with our broad-spectrum diagnostic approach were present in the warts.

The estimated cellular viral loads of HPV2, HPV27, and HPV57 in the caused common warts ranged from 1.12 × 10^0^ to 5.45 × 10^5^ copies per cell, which is in line with similar previous studies [15,20], whereas the cellular viral loads of HPV4 and HPV65 ranged from 1.68 × 10^2^ to 6.75 × 10^5^ copies per cell, and, to our knowledge, were estimated here for the first time in common warts using type-specific qPCR assays. This study showed that the estimated cellular viral loads of HPV4 and HPV65 were significantly higher than the viral loads of HPV2, HPV27, and HPV57 in the caused common warts. The possible reason for the relatively higher estimated viral loads of HPV4 and HPV65 compared with HPVs from the *Alpha*-4 species could be the differential impact of their viral proteins in regulating viral replication, as seen in HPV1, where the viral replication protein E4 is highly abundant in cytoplasmic and nuclear inclusion granules, which supports the initiation of HPV1 genome amplification immediately in the parabasal cell layer of the cutaneous epithelium during the presumed inhibition of cellular DNA replication and cell proliferation in the G2 phase of cell division, resulting in high viral particle synthesis [41,42]. The problem with determining cellular viral loads in tissue biopsies is that a variable amount of adjacent tissue can be included, and HBB from cells in surrounding (non-infected) tissue could give a misleading result [43], even in samples with undetermined causative HPV types. Laser capture micro-dissection in combination with type-specific qPCRs based on the examination of histopathologically strictly selected neoplastic cells from heterogeneous tissue sections would allow a more accurate determination of HPV type-specific viral loads in specific epithelial cell layers [17,44].

In contrast to similar research in Japan [16], a comparison of the patients’ age among the most frequent causative HPV types showed, for the first time to our knowledge, that HPV65 was etiologically associated with the development of common warts in patients with a median age of 52 years, who were significantly older than patients with warts caused by HPV27 and HPV57 (median ages: 22 and 23 years, respectively). Previously, HPV types from the *Gamma*-PV genus have been shown to persist in healthy skin and cause mainly chronic asymptomatic infections associated with long-term viral replication supported by keratinocyte differentiation and viral shedding from the skin surface, minimizing the risk of immune clearance [19,45,46]. It could be possible that the development of HPV65-induced warts in older patients in this cohort was related to a waning immune response (i.e., immunosenescence), similar to that seen in female patients over 50 years of age (48.9% of whom reported a history of an abnormal Pap smear finding and 19.2% reported a history of treatment for cervical intraepithelial neoplasia), in whom low-level persistent high-risk HPV infection is likely to reactivate when immunologic control wanes [47,48].

Whereas HPV4- and HPV65-induced common warts occurred primarily on the hands, HPV2, HPV27, and HPV57 frequently caused warts at various anatomical sites, including the hands, arms, legs, and head. Comparison of the size of common warts at different anatomical sites showed that HPV4 was etiologically associated with the development of significantly smaller common warts than HPV2, HPV27, HPV57, and HPV65. Previously, HPV4 was shown to be associated with the small, endophytic, punctate variant of common warts whose size did not exceed 5 mm, mostly on the hands [49], whereas the median diameter of HPV4-induced warts in this study was 4 mm, ranging from 2 to 10 mm. Following a similar recent study [14], in which the size of skin warts was shown to be a significant feature of wart location regardless of HPV type, only common warts on the hands were included in the additional comparison. In the analysis, it was confirmed that HPV4-induced common warts on the hands were also significantly smaller than the warts caused by HPV2, HPV27, and HPV57.

In this study, HPV2, HPV4, HPV27, HPV57, and HPV65 have been characterized as the five most frequent etiological agents in common warts of immunocompetent patients referred to the tertiary level of dermatological care. This may cause bias, and thus a larger number of wart biopsies obtained across all age groups would probably need to be studied to determine the causative HPV types in common warts in the general population. Nevertheless, data generated in persistent and treatment-resistant common warts could be essential to determine the most appropriate target HPV types for future cutaneous HPV vaccines, which would be particularly beneficial for immunocompromised patients [50]. Further molecular studies of biological differences and immune evasion strategies of cutaneous HPVs are needed to better understand the relatively high cellular viral load of HPV4 and HPV65 in smaller or equal-sized common warts than in HPV2, HPV27, and HPV57, and potential association of HPV65 with the development of warts in older patients. Considering that cutaneous HPVs are part of normal skin microbiota [19], studying the differences in patient demographic characteristics and clinical features of skin warts according to the causative HPV types could be important for understanding the viral pathogenesis of various skin neoplasms, including HPV-related development of cutaneous squamous cell carcinoma [35].

## 5. Conclusions

This study describes an improved diagnostic approach for the comprehensive detection and characterization of causative HPV types in histologically confirmed common warts in immunocompetent patients based on the estimation of HPV type-specific cellular viral loads, thereby importantly improving the knowledge of the diversity and pathogenesis of causative HPV types in common warts. To evaluate the etiological role of all HPV types detected in the development of common warts, we used seven newly developed type-specific qPCR assays targeting eight different HPV types. Using an improved diagnostic strategy to systematically estimate the cellular viral load of each HPV detected and according to the applied cutoff value for determining the causative HPV type, an etiological agent was reliably identified in our main study population in 96.8% of HPV-positive common warts, including several warts with multiple HPV types, whereas the causative HPV types could not be reliably assigned in the four remaining warts. Furthermore, 87.9% of patients from whom multiple samples of common warts were collected had a complete concordance of the causative HPVs identified, confirming that a single HPV type is typically involved in the development of multiple warts in an individual patient. Of the 12 different causative HPV types identified, HPV27 had the highest prevalence rate, followed by HPV57, HPV4, HPV2, and HPV65, which together have been etiologically associated with the development of 89.7% of common warts. The improved diagnostic protocol allowed reliable determination of causative HPV types in the great majority of common warts being HPV DNA-negative or HPV-positive but without a reliably determined causative HPV type in our previous study [15]. To the best of our knowledge, the cellular viral loads of HPV4 and HPV65 from the *Gamma*-1 species were estimated in common warts for the first time in our study and were significantly higher than the viral loads of HPV2, HPV27, and HPV57 from the *Alpha*-4 species. In addition, we showed for the first time that HPV65 was etiologically associated with the development of common warts in significantly older patients than HPV27 and HPV57. HPV2, HPV27, and HPV57 frequently caused common warts at various anatomical sites, whereas HPV4- and HPV65-induced warts primarily occurred on the hands. Furthermore, HPV4-induced warts were significantly smaller than warts caused by HPV2, HPV27, HPV57, and HPV65.

## Figures and Tables

**Table 1:** Primers and double-quenched probes used in quantitative real-time PCR assays.

Primer/Probe	Target	Amplicon Size	Nucleotide Sequence (5′–3′)	Reference
beta-403f	HBB gene	150-bp	TGGGTTTCTGATAGGCACTGACT	[22]
beta-532r			AACAGCATCAGGAGTGGACAGAT	
beta-471pr			FAM-TCTACCCTT-ZEN-GGACCCAGAGGTTCTTTGAGT-IABkFQ	
2–27F			TACCTGCCCCCCAGACATT	[23]
2R	HPV2 (L2 ORF)	144-bp	GGAATGTACCCAGTACGCCC	
HPV2-P0			Cy5-CCCAAGAGT-TAO-GGAACAGAACACTTTAGCA-IAbRQSp	
27R	HPV27 (L2 ORF)	145-bp	AGGAATATACCCGGTACGTCC	
HPV27-P0			HEX-CTAGGGGTC-ZEN-TTCTTTGGCGGTCTTG-IABkFQ	
57F	HPV57 (L2 ORF)	157-bp	GCAAGCAGGCTGGAACG	
57R			GGTATGTAGCCTGTGCGTCC	
HPV57-P0			FAM-TTCGGTGGC-ZEN-CTCGGTATAGGTACT-IABkFQ	
HPV1-forward	HPV1 (L1 ORF)	148-bp	AGCAACATGCAAATATCCTGATT	[24]
HPV1-reverse			TTGTGGGACTGCCTCCTTATC	
HPV1-probe			HEX-GCGAGCAAA-ZEN-TGTATACCAGGCACT-IABkFQ	
HPV63-forward	HPV63 (E2 ORF)	108-bp	TCCTGTCAATAGCTCCCCACT	
HPV63-reverse			GACCCCTTCGTCTCTGCTTT	
HPV63-probe			FAM-ACACCAACC-ZEN-CAGCCACCCAAG-IABkFQ	
HPV4-forward	HPV4 (L2 ORF)	122-bp	TCATATCTGGCACAACCGAAT	This study
HPV4-reverse			AAAGGGGTTCAACGGTTCTAA	
HPV4-probe			FAM-ACATTTTCA-ZEN-GGCGATTCCATTGGTT-IABkFQ	
HPV65-forward	HPV65 (L2 ORF)	130-bp	CCTTTGACGCTGACATCACTC	
HPV65-reverse			ATTTGCCCAGTGTCTGTCTGA	
HPV65-probe			HEX-TATTTGAGC-ZEN-GGGACTTGGAACAGGT-IABkFQ	
HPV3-forward	HPV3 (L2 ORF)	124-bp	TGGATGTGCCTTTACAACCTG	This study
HPV3-reverse			AGATAAAAATCCCCGCCATCT	
HPV3-probe			FAM-CGTTTGTTC-ZEN-CCTTGTCTCCTGTTGA-IABkFQ	
HPV7-forward	HPV7 (E2 ORF)	135-bp	AGCGAAGGAGAGACGGAGACT	This study
HPV7-reverse			GACCTCCACCACTGTTCCTGA	
HPV7-probe			FAM-CGCTCGCCT-ZEN-GATATTGAAAGCAACA-IABkFQ	
HPV10-forward	HPV10 (E6 ORF)	154-bp	GCACAGGAACCCAGAAACATA	This study
HPV10-reverse			CCGCTCTCCACACCAAATATAA	
HPV10-probe			FAM-TGGAATACC-ZEN-TTTGGAGGACCTTCGC-IABkFQ	
HPV28-forward	HPV28 (L2 ORF)	150-bp	AGACATTTGCTTCGCCAGGTA	This study
HPV28-reverse			ATGAGGTGGGACGAGACAAGA	
HPV28-probe			FAM-AGTAGGATC-ZEN-GCTGGACCCCGTCTAT-IABkFQ	
HPV29-forward	HPV29 (L2 ORF)	153-bp	CCCACCGAGGACATAGAGTTG	This study
HPV29-reverse			AGTAATGCGACCCCCGTAAGT	
HPV29-probe			FAM-ATATGCTGA-ZEN-TGTGGACGAGGCTGAC-IABkFQ	
HPV95-forward	HPV95 (E2 ORF)	124-bp	GCTCTACAAACCCCTCCTCCT	This study
HPV95-reverse			GGTTGTAGTTCCCTCGACTGC	
HPV95-probe			FAM-CCCTCCACC-ZEN-AGCAACACCAAAACTA-IABkFQ	

HBB—human beta-globin; HPV—human papillomavirus; ORF—open reading frame; bp—base pairs.

**Table 2:** Prevalence of human papillomavirus (HPV) types in 126 HPV-positive histologically confirmed common warts obtained from 88 immunocompetent patients.

HPV Type	Viral Species	Number of Patients *n* = 88 (%)	Median Age ^a^ Year (Range)	Number of Samples *n* = 126 (%)	Samples with a Single HPV *n* = 88 (%)	Samples with Multiple HPVs *n* = 38 (%)
HPV2	*Alpha*-4	15 (17.0)	25 (15–74)	19 (15.1)	8 (9.1)	11 (28.9)
HPV27		31 (35.2)	23 (10–63)	40 (31.7)	25 (28.4)	15 (39.4)
HPV57		26 (29.5)	24 (9–54)	38 (30.2)	25 (28.4)	13 (34.2)
HPV4	*Gamma*-1	14 (15.9)	23.5 (13–72)	23 (18.3)	15 (17.0)	8 (21.1)
HPV65		15 (17.0)	49 (14–78)	19 (15.1)	8 (9.1)	11 (28.9)
HPV95		1 (1.1)	42	1 (0.8)	–	1 (2.6)
HPV3	*Alpha*-2	1 (1.1)	54	1 (0.8)	–	1 (2.6)
HPV10		3 (3.4)	49 (41–78)	5 (4.0)	2 (2.3)	3 (7.9)
HPV28		1 (1.1)	27	2 (1.6)	2 (2.3)	–
HPV29		1 (1.1)	29	1 (0.8)	1 (1.1)	–
HPV7	*Alpha*-8	1 (1.1)	30	1 (0.8)	1 (1.1)	–
HPV1	*Mu*-1	14 (15.9)	24.5 (15–78)	15 (11.9)	1 (1.1)	14 (36.8)
HPV63	*Mu*-2	2 (2.3)	13, 15	3 (2.4)	–	3 (7.9)

^a^ HPV types that were present in common warts of only one or two patients do not have a calculated median age.

**Table 3:** Prevalence of 12 causative human papillomavirus (HPV) types in 126 HPV-positive histologically confirmed common warts obtained from 88 immunocompetent patients.

Causative HPV Type	Viral Species	Number of Patients *n* = 88 (%) ^a^	Patient Sex ^a^		Median Age ^b^ Year (Range)	Number of Samples *n* = 126 (%)	Caused-Detected Proportion (%) ^c^
			Female *n* = 46 (%)	Male *n* = 42 (%)			
HPV2	*Alpha*-4	14 (15.9)	7 (15.2)	7 (16.7)	24.5 (15–74)	17 (13.5)	17/19 (89.5)
HPV27		25 (28.4)	9 (19.7)	16 (38.1)	22 (10–63)	34 (27.0)	34/40 (85.0)
HPV57		23 (26.1)	13 (28.3)	10 (23.8)	23 (9–54)	33 (26.2)	33/38 (86.8)
HPV4	*Gamma*-1	11 (12.5)	7 (15.2)	4 (9.5)	22 (13–72)	19 (15.1)	19/23 (82.6)
HPV65		7 (8.0)	5 (10.9)	2 (4.8)	52 (15–78)	10 (7.9)	10/19 (52.6)
HPV95		1 (1.1)	1 (2.2)	–	42	1 (0.8)	1/1 (100)
HPV3	*Alpha*-2	1 (1.1)	1 (2.2)	–	54	1 (0.8)	1/1 (100)
HPV10		2 (2.3)	–	2 (4.8)	41, 49	2 (1.6)	2/5 (40.0)
HPV28		1 (1.1)	–	1 (2.4)	27	2 (1.6)	2/2 (100)
HPV29		1 (1.1)	1 (2.2)	–	29	1 (0.8)	1/1 (100)
HPV7	*Alpha*-8	1 (1.1)	1 (2.2)	–	30	1 (0.8)	1/1 (100)
HPV1	*Mu*-1	1 (1.1)	1 (2.2)	–	45	1 (0.8)	1/15 (6.7)
HPV63	*Mu*-2	–	–	–	–	–	0/3 (0.0)
Undetermined	–	4 (4.5)	1 (2.2)	3 (7.1)	54.5 (41–62)	4 (3.2)	–

^a^ Three patients with multiple samples of common warts had different causative HPV types in different warts (Patients nos. 14, 35, and 52; Table S1), whereas one patient had one wart with a determined causative HPV type and one with an undetermined causative HPV type (Patient no. 89; Table S1); ^b^ HPV types that caused common warts in only one or two patients do not have a calculated median age; ^c^ Proportion of common warts caused by HPV according to the warts in which this HPV was detected.

**Table 4:** Minimum, maximum, median, mean, and standard deviation of estimated cellular viral loads of the five most frequent causative human papillomavirus (HPV) types (HPV2, HPV27, HPV57, HPV4, and HPV65) detected in 113 histologically confirmed common warts obtained from 77 immunocompetent patients.

Causative HPV Type	Number of Samples *n* = 113	Viral Load (Viral Copies/Cell)				
		Minimum	Maximum	Median	Mean	Standard Deviation
HPV2	17	1.12 × 10^0^	5.45 × 10^5^	6.73 × 10^3^	4.28 × 10^4^	1.30 × 10^5^
HPV27	34	1.85 × 10^0^	1.78 × 10^5^	7.73 × 10^3^	1.71 × 10^4^	3.05 × 10^4^
HPV57	33	5.23 × 10^0^	4.97 × 10^4^	8.65 × 10^3^	1.35 × 10^4^	1.38 × 10^4^
HPV4	19	1.68 × 10^2^	5.65 × 10^5^	2.75 × 10^4^	9.19 × 10^4^	1.68 × 10^5^
HPV65	10	1.53 × 10^4^	6.75 × 10^5^	1.64 × 10^5^	2.00 × 10^5^	1.82 × 10^5^

## Data Availability

Not applicable.

## Supplementary Materials

The following supporting information can be downloaded at: https://www.mdpi.com/article/10.3390/v14102266/s1, Table S1: Characteristics of 90 immunocompetent patients and 128 corresponding histologically confirmed common warts with the estimated cellular viral loads of human papillomavirus (HPV) types detected and causative HPV types determined; Table S2: Etiological characterization of nine common wart samples in which human papillomavirus (HPV) DNA was not detected in our previous study [15]; Table S3: Etiological characterization of 25 common wart samples in which the causative human papillomavirus (HPV) types could not be reliably determined in our previous study [15].

## References

[1] Doorbar J., Quint W., Banks L., Bravo I.G., Stoler M., Broker T.R., Stanley M.A. (2012). The biology and life-cycle of human papillomaviruses. Vaccine.

[2] International Human Papillomavirus (HPV) Reference Center—HPV Reference Clones. https://www.hpvcenter.se/human_reference_clones/.

[3] Bruggink S.C., Gussekloo J., de Koning M.N., Feltkamp M.C., Bavinck J.N., Quint W.G., Assendelft W.J., Eekhof J.A. (2013). HPV type in plantar warts influences natural course and treatment response: Secondary analysis of a randomised controlled trial. J. Clin. Virol..

[4] Hogendoorn G.K., Bruggink S.C., de Koning M.N.C., Eekhof J.A.H., Hermans K.E., Rissmann R., Burggraaf J., Wolterbeek R., Quint K.D., Kouwenhoven S.T.P. (2018). Morphological characteristics and human papillomavirus genotype predict the treatment response in cutaneous warts. Br. J. Dermatol..

[5] Cardoso J.C., Calonje E. (2011). Cutaneous manifestations of human papillomaviruses: A review. Acta Dermatovenerol. Alp. Pannonica Adriat..

[6] Bacaj P., Burch D. (2018). Human papillomavirus infection of the skin. Arch. Pathol. Lab. Med..

[7] Rübben A., Kalka K., Spelten B., Grussendorf-Conen E.I. (1997). Clinical features and age distribution of patients with HPV 2/27/57-induced common warts. Arch. Dermatol. Res..

[8] Harwood C.A., Spink P.J., Surentheran T., Leigh I.M., de Villiers E.M., McGregor J.M., Proby C.M., Breuer J. (1999). Degenerate and nested PCR: A highly sensitive and specific method for detection of human papillomavirus infection in cutaneous warts. J. Clin. Microbiol..

[9] Porro A.M., Alchorne M.M., Mota G.R., Michalany N., Pignatari A.C., Souza I.E. (2003). Detection and typing of human papillomavirus in cutaneous warts of patients infected with human immunodeficiency virus type 1. Br. J. Dermatol..

[10] Lei Y.J., Gao C., Wang C., Han J., Chen J.M., Xiang G.C., Shi Q., Jiang H.Y., Zhou W., An R. (2009). Molecular epidemiological study on prevalence of human papillomaviruses in patients with common warts in Beijing area. Biomed. Environ. Sci..

[11] Bruggink S.C., de Koning M.N., Gussekloo J., Egberts P.F., Ter Schegget J., Feltkamp M.C., Bavinck J.N., Quint W.G., Assendelft W.J., Eekhof J.A. (2012). Cutaneous wart-associated HPV types: Prevalence and relation with patient characteristics. J. Clin. Virol..

[12] Al Bdour S., Akkash L., Shehabi A.A. (2013). Detection and typing of common human papillomaviruses among Jordanian patients. J. Med. Virol..

[13] Giannaki M., Kakourou T., Theodoridou M., Syriopoulou V., Kabouris M., Louizou E., Chrousos G. (2013). Human papillomavirus (HPV) genotyping of cutaneous warts in Greek children. Pediatr. Dermatol..

[14] Al-Awadhi R., Al-Mutairi N., Chehadeh W. (2020). Prevalence of HPV genotypes in adult male patients with cutaneous warts: A cross-sectional study. Med. Princ. Pract..

[15] Breznik V., Fujs Komloš K., Hošnjak L., Luzar B., Kavalar R., Miljković J., Poljak M. (2020). Determination of causative human papillomavirus type in tissue specimens of common warts based on estimated viral loads. Front. Cell. Infect. Microbiol..

[16] Hagiwara K., Uezato H., Arakaki H., Nonaka S., Nonaka K., Nonaka H., Asato T., Oshiro M., Kariya K., Hattori A. (2005). A genotype distribution of human papillomaviruses detected by polymerase chain reaction and direct sequencing analysis in a large sample of common warts in Japan. J. Med. Virol..

[17] Quint W., Jenkins D., Molijn A., Struijk L., van de Sandt M., Doorbar J., Mols J., Van Hoof C., Hardt K., Struyf F. (2012). One virus, one lesion—Individual components of CIN lesions contain a specific HPV type. J. Pathol..

[18] Forslund O., Lindelöf B., Hradil E., Nordin P., Stenquist B., Kirnbauer R., Slupetzky K., Dillner J. (2004). High prevalence of cutaneous human papillomavirus DNA on the top of skin tumors but not in “stripped” biopsies from the same tumors. J. Investig. Dermatol..

[19] Foulongne V., Sauvage V., Hebert C., Dereure O., Cheval J., Gouilh M.A., Pariente K., Segondy M., Burguière A., Manuguerra J.C. (2012). Human skin microbiota: High diversity of DNA viruses identified on the human skin by high throughput sequencing. PLoS ONE.

[20] Köhler A., Meyer T., Stockfleth E., Nindl I. (2009). High viral load of human wart-associated papillomaviruses (PV) but not beta-PV in cutaneous warts independent of immunosuppression. Br. J. Dermatol..

[21] Tom L.N., Dix C.F., Hoang V.L.T., Lin L.L., Nufer K.L., Tomihara S., Prow N.A., Soyer H.P., Prow T.W., Ardigo M. (2016). Skin microbiopsy for HPV DNA detection in cutaneous warts. J. Eur. Acad. Dermatol. Venereol..

[22] van Duin M., Snijders P.J., Schrijnemakers H.F., Voorhorst F.J., Rozendaal L., Nobbenhuis M.A., van den Brule A.J., Verheijen R.H., Helmerhorst T.J., Meijer C.J. (2002). Human papillomavirus 16 load in normal and abnormal cervical scrapes: An indicator of CIN II/III and viral clearance. Int. J. Cancer.

[23] Hošnjak L., Fujs Komloš K., Kocjan B.J., Seme K., Poljak M. (2016). Development of a novel multiplex type-specific quantitative real-time PCR for detection and differentiation of infections with human papillomavirus types HPV2, HPV27, and HPV57. Acta Dermatovenerol. Alp. Pannonica Adriat..

[24] Šterbenc A., Hošnjak L., Chouhy D., Bolatti E.M., Oštrbenk A., Seme K., Kocjan B.J., Luzar B., Giri A.A., Poljak M. (2017). Molecular characterization, tissue tropism, and genetic variability of the novel Mupapillomavirus type HPV204 and phylogenetically related types HPV1 and HPV63. PLoS ONE.

[25] Stephenson F.H. (2011). Chapter 9—The real-time polymerase chain reaction (RT-PCR). Calculations for Molecular Biology and Biotechnology: A Guide to Mathematics in the Laboratory.

[26] Chouhy D., Bolatti E.M., Piccirilli G., Sánchez A., Fernandez Bussy R., Giri A.A. (2013). Identification of human papillomavirus type 156, the prototype of a new human gammapapillomavirus species, by a generic and highly sensitive PCR strategy for long DNA fragments. J. Gen. Virol..

[27] Odar K., Kocjan B.J., Hošnjak L., Gale N., Poljak M., Zidar N. (2014). Verrucous carcinoma of the head and neck—not a human papillomavirus-related tumour. J. Cell. Mol. Med..

[28] Hošnjak L., Kocjan B.J., Pirš B., Seme K., Poljak M. (2015). Characterization of two novel gammapapillomaviruses, HPV179 and HPV184, isolated from common warts of a renal-transplant recipient. PLoS ONE.

[29] Platt A.R., Woodhall R.W., George A.L. (2007). Improved DNA sequencing quality and efficiency using an optimized fast cycle sequencing protocol. Biotechniques.

[30] Kocjan B.J., Poljak M., Seme K., Potocnik M., Fujs K., Babic D.Z. (2005). Distribution of human papillomavirus genotypes in plucked eyebrow hairs from Slovenian males with genital warts. Infect. Genet. Evol..

[31] Koressaar T., Remm M. (2007). Enhancements and modifications of primer design program Primer3. Bioinformatics.

[32] Untergasser A., Cutcutache I., Koressaar T., Ye J., Faircloth B.C., Remm M., Rozen S.G. (2012). Primer3—New capabilities and interfaces. Nucleic Acids Res..

[33] Van Doorslaer K., Li Z., Xirasagar S., Maes P., Kaminsky D., Liou D., Sun Q., Kaur R., Huyen Y., McBride A.A. (2017). The Papillomavirus Episteme: A major update to the papillomavirus sequence database. Nucleic Acids Res..

[34] Bae J.M., Kang H., Kim H.O., Park Y.M. (2009). Differential diagnosis of plantar wart from corn, callus and healed wart with the aid of dermoscopy. Br. J. Dermatol..

[35] Aldabagh B., Angeles J.G., Cardones A.R., Arron S.T. (2013). Cutaneous squamous cell carcinoma and human papillomavirus: Is there an association?. Dermatol. Surg..

[36] García-Oreja S., Álvaro-Afonso F.J., Sevillano-Fernández D., Tardáguila-García A., López-Moral M., Lázaro-Martínez J.L. (2022). A non-invasive method for diagnosing plantar warts caused by human papillomavirus (HPV). J. Med. Virol..

[37] de Koning M.N., Quint K.D., Bruggink S.C., Gussekloo J., Bouwes Bavinck J.N., Feltkamp M.C., Quint W.G., Eekhof J.A. (2015). High prevalence of cutaneous warts in elementary school children and the ubiquitous presence of wart-associated human papillomavirus on clinically normal skin. Br. J. Dermatol..

[38] Iftner A., Klug S.J., Garbe C., Blum A., Stancu A., Wilczynski S.P., Iftner T. (2003). The prevalence of human papillomavirus genotypes in nonmelanoma skin cancers of nonimmunosuppressed individuals identifies high-risk genital types as possible risk factors. Cancer Res..

[39] Doorbar J., Egawa N., Griffin H., Kranjec C., Murakami I. (2015). Human papillomavirus molecular biology and disease association. Rev. Med. Virol..

[40] Egawa K., Kimmel R., De Villiers E.M. (2005). A novel type of human papillomavirus (HPV 95): Comparison with infections of closely related human papillomavirus types. Br. J. Dermatol..

[41] Doorbar J. (2013). The E4 protein; structure, function and patterns of expression. Virology.

[42] Ghorzang E., de Koning M.N.C., Bouwes Bavinck J.N., Gussekloo J., Quint K.D., Goeman J.J., Feltkamp M.C.W., Bruggink S.C., Eekhof J.A.H. (2022). HPV type-specific distribution among family members and linen in households of cutaneous wart patients. J. Eur. Acad. Dermatol. Venereol..

[43] Malin K., Louise B.M., Gisela H., Mats K.G., Gabriella L.L. (2021). Optimization of droplet digital PCR assays for the type-specific detection and quantification of five HPV genotypes, including additional data on viral loads of nine different HPV genotypes in cervical carcinomas. J. Virol. Methods.

[44] Lebelo R.L., Thys S., Benoy I., Depuydt C.E., Bogers J.P., Bida M.N., Mphahlele M.J. (2015). Laser micro-dissection and qPCR for identifying specific HPV types responsible for malignancy in penile lesions. J. Med. Virol..

[45] Hazard K., Karlsson A., Andersson K., Ekberg H., Dillner J., Forslund O. (2007). Cutaneous human papillomaviruses persist on healthy skin. J. Investig. Dermatol..

[46] Egawa N., Egawa K., Griffin H., Doorbar J. (2015). Human Papillomaviruses; Epithelial Tropisms, and the Development of Neoplasia. Viruses.

[47] Brown D.R., Weaver B. (2013). Human papillomavirus in older women: New infection or reactivation?. J. Infect. Dis..

[48] Gravitt P.E., Rositch A.F., Silver M.I., Marks M.A., Chang K., Burke A.E., Viscidi R.P. (2013). A cohort effect of the sexual revolution may be masking an increase in human papillomavirus detection at menopause in the United States. J. Infect. Dis..

[49] Jablonska S., Orth G., Obalek S., Croissant O. (1985). Cutaneous warts. Clinical, histologic, and virologic correlations. Clin. Dermatol..

[50] Gaiser M.R., Textor S., Senger T., Schädlich L., Waterboer T., Kaufmann A.M., Süsal C., Pawlita M., Enk A.H., Gissmann L. (2015). Evaluation of specific humoral and cellular immune responses against the major capsid L1 protein of cutaneous wart-associated alpha-Papillomaviruses in solid organ transplant recipients. J. Dermatol. Sci..

