# Supplementary material for: An Improved Protocol for Comprehensive Etiological Characterization of Skin Warts and Determining Causative Human Papillomavirus Types in 128 Histologically Confirmed Common Warts
Source: Viruses. 2022 Oct 15;14(10):2266. doi: 10.3390/v14102266 (PMC9612167; doi:10.3390/v14102266)
Supplement: Supplementary file 1 [file viruses-14-02266-s001.zip › viruses-1945441-supplementary.pdf]

SUPPLEMENTARY MATERIALS

An improved protocol for comprehensive etiological characterization of skin warts and determining causative human papillomavirus types in 128 histologically confirmed common warts

Table S1: Characteristics of 90 immunocompetent patients and 128 corresponding histologically confirmed common warts with the estimated cellular viral loads of human papillomavirus (HPV) types detected and causative HPV types determined.

Sample no.	Patient no.	Patient sex	Patient age (year)	Wart location	Wart duration (month)	Wart diameter (mm)	HPV2 VL (copies/cell)	HPV27 VL (copies/cell)	HPV57 VL (copies/cell)	HPV4 VL (copies/cell)	HPV65 VL (copies/cell)	Other HPV VL (copies/cell)	Causative HPV type [a]
1	1	F	17	R hand	3	8	-	-	**1.49×10^{4}**	-	-	-	HPV57
2	2a	M	31	head	4	8	-	-	**2.88×10^{3}**	-	-	-	HPV57
3	2b	M	31	head	2	3	-	-	**1.04×10^{3}**	-	-	-	HPV57
4	3	M	14	R leg	12	15	-	**1.16×10^{4}**	-	-	-	-	HPV27
5	4	F	14	L leg	12	13	-	**1.78×10^{3}**	-	-	-	-	HPV27
6	5	M	38	R hand	36	6	-	-	**1.59×10^{4}**	-	-	-	HPV57
7	6	M	16	head	12	3	-	-	**7.31×10^{2}**	-	-	-	HPV57
8	7	F	16	L hand	24	10	-	**1.78×10^{5}**	-	-	-	-	HPV27
9	8	M	15	R hand	12	7	**1.24×10^{4}**	-	-	-	-	HPV1: 8.92×10^{-4}	HPV2
10	9	F	33	L hand	6	5	**1.44×10^{4}**	-	-	-	-	-	HPV2
11	10	M	23	R hand	12	4	-	**1.52×10^{4}**	-	-	-	-	HPV27
12	11	M	49	L arm	24	15	-	-	-	-	-	HPV10: **6.07×10^{3}**	HPV10
13	12	F	25	R hand	60	4	**5.35×10^{4}**	-	-	-	1.25×10^{-2}	-	HPV2
14	13	F	29	R hand	24	8	-	-	**1.16×10^{4}**	-	-	-	HPV57
15	14a	M	22	R hand	12	2	2.01×10^{-3}	-	-	**1.68×10^{2}**	-	-	HPV4
16	14b	M	22	R hand	1	8	**1.99×10^{4}**	-	-	6.40×10^{-4}	-	-	HPV2
17	15	M	22	L leg	6	9	**3.63×10^{4}**	-	-	-	-	-	HPV2
18	16a	F	20	L hand	24	10	-	-	-	**5.49×10^{5}**	-	-	HPV4
19	16b	F	20	L hand	24	4	-	-	-	**2.75×10^{4}**	-	-	HPV4
20	17	F	30	R hand	36	4	-	-	-	-	-	HPV7: **1.75×10^{1}**	HPV7
21	18a	F	78	R hand	2	8	-	-	-	-	**1.42×10^{5}**	HPV10: 8.97×10^{-3}	HPV65

[a] Viral loads of causative HPV types are indicated in bold; F, female; M, male; R, right; L, left; VL, viral load; WD, common wart without determined causative HPV type.

Table S1. *Cont.*

Sample no.	Patient no.	Patient sex	Patient age (year)	Wart location	Wart duration (month)	Wart diameter (mm)	HPV2 VL (copies/cell)	HPV27 VL (copies/cell)	HPV57 VL (copies/cell)	HPV4 VL (copies/cell)	HPV65 VL (copies/cell)	Other HPV VL (copies/cell)	Causative HPV type [a]
22	18b	F	78	R hand	2	11	-	-	-	-	**1.08×10^{5}**	HPV1: 6.05×10^{-2}; HPV10: 9.48×10^{-3}	HPV65
23	19	M	70	R hand	10	6	-	-	-	-	**6.75×10^{5}**	-	HPV65
24	20a	M	17	head	6	3	-	-	**3.11×10^{3}**	-	-	-	HPV57
25	20b	M	17	R hand	18	3	-	-	**1.32×10^{4}**	-	-	-	HPV57
26	21	M	28	L hand	24	5	-	-	**4.33×10^{3}**	-	-	-	HPV57
27	22	M	16	L arm	3	3	**8.56×10^{1}**	1.14×10^{-1}	-	-	-	HPV1: 6.30×10^{-3}	HPV2
28	23	M	54	R hand	18	6	-	**1.85×10^{0}**	-	-	2.39×10^{-2}	-	HPV27
29	24	F	50	R leg	6	7	-	-	-	-	8.65×10^{-5}	-	WD
30	25	F	54	L arm	36	7	-	-	-	-	2.23×10^{-3}	HPV3: **2.17×10^{4}**	HPV3
31	26	F	29	L hand	36	20	-	-	-	-	-	HPV29: **1.02×10^{4}**	HPV29
32	27	F	46	L leg	72	10	-	**3.24×10^{4}**	-	-	-	-	HPV27
33	28	M	29	R hand	60	3	-	-	**5.23×10^{0}**	-	-	-	HPV57
34	29a	M	15	R hand	36	7	-	-	**2.13×10^{4}**	-	-	HPV63: 4.16×10^{-4}	HPV57
35	29b	M	15	R hand	36	7	-	-	**2.33×10^{4}**	-	-	-	HPV57
36	29c	M	15	head	3	5	-	-	**3.03×10^{3}**	-	-	HPV63: 3.42×10^{-4}	HPV57
37	30	F	9	L leg	12	8	-	-	**3.29×10^{4}**	-	-	-	HPV57
38	31	F	20	R arm	3	9	**1.34×10^{4}**	-	-	-	-	HPV1: 1.67×10^{-1}	HPV2
39	32a	F	16	R hand	48	5	-	-	**6.06×10^{2}**	-	-	-	HPV57
40	32b	F	16	L hand	48	5	-	-	**2.30×10^{4}**	-	-	-	HPV57
41	33	M	20	R hand	12	5	-	**2.45×10^{4}**	-	3.25×10^{-4}	-	HPV1: 9.77×10^{-3}	HPV27
42	34a	F	49	R hand	12	6	-	-	-	-	**2.66×10^{5}**	-	HPV65
43	34b	F	49	R hand	12	3	-	-	-	-	**2.03×10^{5}**	-	HPV65
44	35a	M	15	R hand	12	7	-	-	-	-	**1.12×10^{5}**	-	HPV65
45	35b	M	15	R hand	12	3	-	-	-	**4.45×10^{3}**	-	-	HPV4
46	35c	M	15	L hand	24	3	-	-	-	**1.61×10^{4}**	-	-	HPV4
47	36a	F	35	L hand	18	13	-	2.84×10^{-2}	**2.84×10^{4}**	-	4.29×10^{-4}	-	HPV57
48	36b	F	35	L hand	12	4	-	-	**4.87×10^{3}**	-	3.13×10^{-4}	-	HPV57
49	37a	F	13	L hand	48	7	-	-	-	**1.29×10^{5}**	-	HPV63: 6.19×10^{-4}	HPV4
50	37b	F	13	L hand	36	4	-	-	-	**1.01×10^{5}**	-	-	HPV4

[a] Viral loads of causative HPV types are indicated in bold; F, female; M, male; R, right; L, left; VL, viral load; WD, common wart without determined causative HPV type.

Table S1. *Cont.*

Sample no.	Patient no.	Patient sex	Patient age (year)	Wart location	Wart duration (month)	Wart diameter (mm)	HPV2 VL (copies/cell)	HPV27 VL (copies/cell)	HPV57 VL (copies/cell)	HPV4 VL (copies/cell)	HPV65 VL (copies/cell)	Other HPV VL (copies/cell)	Causative HPV type [a]
51	37c	F	13	R hand	24	4	-	-	-	**3.25×10^{4}**	-	-	HPV4
52	38	F	23	R leg	18	9	-	-	**3.67×10^{3}**	-	-	HPV1: 1.30×10^{-1}	HPV57
53	39	M	13	head	1	3	-	**1.45×10^{4}**	-	-	-	-	HPV27
54	40	F	51	R hand	6	7	-	**2.00×10^{4}**	-	-	-	-	HPV27
55	41	F	17	head	3	4	-	-	**7.17×10^{3}**	-	1.95×10^{-4}	-	HPV57
56	42	M	34	L leg	6	5	-	-	**2.18×10^{4}**	-	-	HPV1: 3.16×10^{-1}	HPV57
57	43a	M	22	R hand	18	10	-	**5.87×10^{2}**	-	-	-	HPV1: 3.61×10^{-2}	HPV27
58	43b	M	22	R hand	12	3	-	**8.01×10^{3}**	-	-	-	-	HPV27
59	43c	M	22	R hand	12	4	-	**2.36×10^{4}**	-	-	-	-	HPV27
60	44a	M	25	L leg	3	5	-	**9.47×10^{3}**	-	-	-	HPV1: 6.73×10^{-2}	HPV27
61	44b	M	25	R hand	3	7	-	**3.78×10^{3}**	-	-	-	HPV1: 3.50×10^{-1}	HPV27
62	45a	F	22	R arm	48	5	-	-	**4.97×10^{4}**	-	-	-	HPV57
63	45b	F	22	R arm	24	4	-	-	**1.24×10^{4}**	-	-	-	HPV57
64	46a	F	43	R hand	36	8	-	**5.42×10^{3}**	-	-	-	-	HPV27
65	46b	F	43	R hand	6	4	-	**4.46×10^{4}**	-	-	-	-	HPV27
66	47	F	52	arm	60	6	-	-	2.97×10^{-3}	-	**2.18×10^{5}**	-	HPV65
67	48	M	19	L arm	36	11	-	**3.20×10^{3}**	-	-	-	-	HPV27
68	49a	F	40	hand	2	4	-	-	-	-	**7.60×10^{4}**	-	HPV65
69	49b	F	40	hand	2	5	-	-	-	-	**1.85×10^{5}**	-	HPV65
70	50a	M	21	L hand	12	6	-	**1.46×10^{4}**	-	-	-	-	HPV27
71	50b	M	21	L hand	1	8	-	**1.00×10^{4}**	-	-	1.11×10^{-2}	-	HPV27
72	51a	M	21	L hand	18	5	-	-	-	**2.43×10^{4}**	-	-	HPV4
73	51b	M	21	L hand	18	4	-	-	-	**1.13×10^{4}**	-	-	HPV4
74	52a	F	25	R hand	12	2	-	-	**8.06×10^{1}**	-	-	-	HPV57
75	52b	F	25	L hand	8	3	-	-	1.39×10^{-3}	**2.35×10^{4}**	-	-	HPV4
76	53a	F	36	R hand	12	3	-	**1.21×10^{3}**	-	-	-	-	HPV27
77	53b	F	36	R hand	12	4	-	**1.23×10^{3}**	-	-	-	HPV1: 1.65×10^{-4}	HPV27
78	54	M	62	L hand	60	6	-	-	-	3.23×10^{-3}	-	-	WD
79	55	F	20	R hand	2	3	-	-	-	**2.98×10^{2}**	-	-	HPV4
80	56	F	69	R hand	12	4	-	-	-	-	**1.53×10^{4}**	-	HPV65
81	57	F	13	L leg	6	3	-	-	**1.74×10^{2}**	-	-	-	HPV57

[a] Viral loads of causative HPV types are indicated in bold; F, female; M, male; R, right; L, left; VL, viral load; WD, common wart without determined causative HPV type.

Table S1. *Cont.*

Sample no.	Patient no.	Patient sex	Patient age (year)	Wart location	Wart duration (month)	Wart diameter (mm)	HPV2 VL (copies/cell)	HPV27 VL (copies/cell)	HPV57 VL (copies/cell)	HPV4 VL (copies/cell)	HPV65 VL (copies/cell)	Other HPV VL (copies/cell)	Causative HPV type [a]
82	58	M	16	R arm	36	5	-	**6.00×10^{3}**	-	-	-	-	HPV27
83	59a	M	10	R arm	12	4	-	**3.18×10^{4}**	-	-	-	-	HPV27
84	59b	M	10	R arm	12	4	-	**4.77×10^{3}**	-	-	-	-	HPV27
85	60	M	14	R hand	24	4	-	**3.03×10^{2}**	-	-	1.29×10^{-4}	-	HPV27
86	61a	F	36	R hand	12	6	-	**6.93×10^{3}**	-	-	-	-	HPV27
87	61b	F	36	R hand	12	3	-	**3.32×10^{3}**	-	-	-	-	HPV27
88	62	F	63	L hand	6	6	**5.16×10^{3}**	-	-	-	-	-	HPV2
89	63a	F	56	R hand	12	4	-	-	-	**8.85×10^{2}**	-	-	HPV4
90	63b	F	56	R hand	12	5	-	-	-	**7.16×10^{3}**	-	-	HPV4
91	64	M	24	L hand	3	6	**1.42×10^{4}**	-	-	-	-	HPV1: 1.31×10^{-4}	HPV2
92	65	M	63	R hand	12	8	-	**3.23×10^{4}**	-	-	-	-	HPV27
93	66a	M	25	R hand	6	4	-	5.82×10^{-2}	-	**6.40×10^{4}**	-	-	HPV4
94	66b	M	25	R hand	6	4	4.35×10^{-2}	-	-	**5.65×10^{5}**	-	-	HPV4
95	66c	M	25	R hand	6	4	-	-	-	**4.97×10^{4}**	-	-	HPV4
96	67	F	42	R hand	24	5	-	2.48×10^{-2}	-	-	-	HPV95: **5.87×10^{2}**	HPV95
97	68a	F	52	R hand	24	11	-	-	**3.35×10^{4}**	-	-	-	HPV57
98	68b	F	52	R hand	24	5	-	-	**8.65×10^{3}**	-	-	-	HPV57
99	69	M	54	L hand	12	10	-	-	**2.73×10^{4}**	-	-	-	HPV57
100	70	F	72	R hand	12	4	-	-	-	**8.49×10^{4}**	-	-	HPV4
101	71	M	20	L leg	3	8	-	-	**1.70×10^{4}**	-	-	-	HPV57
102	72	F	34	R hand	1	3	-	-	-	**5.48×10^{4}**	-	-	HPV4
103	73	F	45	L hand	3	6	-	-	-	-	-	HPV1: **2.53×10^{4}**	HPV1
104	74	F	32	R hand	5	6	-	**5.16×10^{3}**	-	-	-	-	HPV27
105	75	F	74	R hand	24	8	**1.12×10^{0}**	-	-	-	-	-	HPV2
106	76	M	21	R hand	4	6	-	**5.19×10^{3}**	-	-	-	-	HPV27
107	77	F	36	L hand	120	5	-	-	-	-	-	-	WD
108	78a	M	26	R hand	24	6	-	**2.48×10^{4}**	-	1.27×10^{-4}	-	-	HPV27
109	78b	M	26	L hand	12	4	-	**6.33×10^{3}**	-	-	-	-	HPV27
110	79	M	48	R leg	12	5	-	-	-	-	-	-	WD
111	80a	M	36	R hand	36	12	**6.73×10^{3}**	-	-	-	-	-	HPV2
112	80b	M	36	R hand	12	5	**1.31×10^{3}**	-	-	-	-	-	HPV2

[a] Viral loads of causative HPV types are indicated in bold; F, female; M, male; R, right; L, left; VL, viral load; WD, common wart without determined causative HPV type.

Table S1. *Cont.*

Sample no.	Patient no.	Patient sex	Patient age (year)	Wart location	Wart duration (month)	Wart diameter (mm)	HPV2 VL (copies/cell)	HPV27 VL (copies/cell)	HPV57 VL (copies/cell)	HPV4 VL (copies/cell)	HPV65 VL (copies/cell)	Other HPV VL (copies/cell)	Causative HPV type [a]
113	81a	F	23	R hand	2	10	**5.45×10^5**	-	-	-	-	-	HPV2
114	81b	F	23	L arm	2	10	**2.46×10^3**	-	9.52×10^{-3}	-	-	-	HPV2
115	82a	M	27	R hand	12	5	-	-	-	-	-	HPV28: **2.14×10^5**	HPV28
116	82b	M	27	R hand	12	10	-	-	-	-	-	HPV28: **6.21×10^5**	HPV28
117	83a	F	15	L hand	6	7	-	-	**4.93×10^4**	-	-	-	HPV57
118	83b	F	15	L arm	6	3	-	5.01×10^{-4}	**2.13×10^2**	-	-	-	HPV57
119	84	F	22	L hand	2	2	-	**7.46×10^3**	-	-	-	-	HPV27
120	85	M	31	R hand	12	10	-	**2.25×10^4**	-	-	-	-	HPV27
121	86a	F	49	L hand	120	6	-	-	**7.38×10^3**	-	-	-	HPV57
122	86b	F	49	L hand	60	13	-	-	**3.03×10^3**	-	-	-	HPV57
123	87a	M	41	R hand	6	7	**2.29×10^3**	-	6.38×10^{-4}	-	-	-	HPV2
124	87b	M	41	R hand	5	4	**2.59×10^1**	-	5.04×10^{-2}	-	-	-	HPV2
125	88	M	59	R hand	120	12	-	8.13×10^{-3}	-	-	-	HPV1: 7.61×10^{-2}	WD
126	89a	M	41	R hand	24	7	-	-	-	-	-	HPV10: **1.01×10^3**	HPV10
127	89b	M	41	R hand	6	5	-	-	-	-	-	HPV1: 1.12×10^{-3}; HPV10: 1.09×10^{-1}	WD
128	90	F	25	R hand	8	3	**1.86×10^1**	-	-	-	-	-	HPV2

[a] Viral loads of causative HPV types are indicated in bold; F, female; M, male; R, right; L, left; VL, viral load; WD, common wart without determined causative HPV type.

Table S2: Etiological characterization of nine common wart samples in which human papillomavirus (HPV) DNA was not detected in our previous study [15].

Sample no.	Sample code [15]	Viral load (viral copies/cell)		Causative HPV type [a]
		HPV4	HPV65	
1	CW148A	-	**1.58×10^{5}**	HPV65
2	CW189B	**5.37×10^{5}**	-	HPV4
3	CW189C	8.03×10^{-1}	-	WD
4	CW201A	-	**2.87×10^{4}**	HPV65
5	CW235	-	1.70×10^{-4}	WD
6	CW253B	-	**2.54×10^{5}**	HPV65
7	CW255A	**1.98×10^{5}**	-	HPV4
8	CW255B	**2.64×10^{3}**	-	HPV4
9	CW255C	**8.82×10^{3}**	-	HPV4

[a] Viral loads of causative HPV types are indicated in bold; WD, common wart without determined causative HPV type.

Table S3: Etiological characterization of 25 common wart samples in which the causative human papillomavirus (HPV) types could not be reliably determined in our previous study [15].

Sample no.	Sample code [15]	Viral load (viral copies/cell) - previously estimated [15]					Viral load (viral copies/cell) - estimated in this study					Causative HPV type [a]
		HPV2	HPV27	HPV57	HPV1	HPV63	HPV4	HPV65	HPV7	HPV10	HPV29	
1	CW040B	-	-	-	2.6×10^{-2}	-	-	1.44×10^{-4}	-	-	-	WD
2	CW078C	-	3.8×10^{-3}	-	2.1×10^{-3}	-	**5.12×10^{4}**	-	-	-	-	HPV4
3	CW083A	-	1.0×10^{-1}	-	6.1×10^{-3}	-	-	-	-	-	-	WD
4	CW094C	-	2.6×10^{-3}	6.0×10^{-4}	-	-	**4.58×10^{4}**	-	-	-	-	HPV4
5	CW114A	2.5×10^{-3}	-	-	-	-	**1.10×10^{2}**	-	-	-	-	HPV4
6	CW117	-	-	-	4.9×10^{-2}	-	-	-	**9.87×10^{3}**	-	-	HPV7
7	CW141A	-	-	1.0×10^{-3}	-	-	**3.98×10^{5}**	-	-	-	-	HPV4
8	CW141B	-	-	-	-	1.5×10^{-3}	**2.40×10^{4}**	-	-	-	-	HPV4
9	CW171B	-	-	1.9×10^{-3}	-	-	-	-	**1.20×10^{1}**	-	-	HPV7
10	CW189A	-	-	7.0×10^{-4}	3.0×10^{-3}	-	**6.79×10^{4}**	-	-	-	-	HPV4
11	CW201B	-	-	-	-	1.1×10^{-3}	-	**1.36×10^{5}**	-	-	-	HPV65
12	CW201C	-	-	-	5.7×10^{-3}	2.1×10^{-3}	-	**1.07×10^{5}**	-	4.10×10^{-3}	-	HPV65
13	CW206	-	-	-	4.6×10^{-3}	-	-	-	**1.64×10^{4}**	-	-	HPV7
14	CW207A	-	-	7.0×10^{-4}	-	-	-	2.59×10^{-4}	-	-	-	WD
15	CW209A	-	-	-	2.1×10^{-2}	-	2.27×10^{-3}	-	-	-	-	WD
16	CW215A	-	-	6.0×10^{-4}	-	-	-	1.79×10^{-4}	-	-	-	WD
17	CW223B	-	-	-	2.5×10^{-2}	-	-	**1.55×10^{5}**	-	1.35×10^{-2}	-	HPV65
18	CW223C	-	-	-	3.3×10^{-3}	-	-	-	-	**1.65×10^{2}**	-	HPV10
19	CW239A	-	-	-	1.7×10^{-3}	-	-	-	-	-	**1.15×10^{4}**	HPV29
20	CW253A	-	-	-	8.2×10^{-3}	-	-	**4.65×10^{5}**	-	-	-	HPV65
21	CW254A	-	-	-	5.0×10^{-4}	-	-	9.79×10^{-5}	-	-	-	WD
22	CW260A	-	-	-	1.6×10^{-2}	6.6×10^{-3}	**1.60×10^{5}**	-	-	-	-	HPV4
23	CW260B	-	-	-	1.4×10^{-1}	1.1×10^{-2}	**6.57×10^{4}**	-	-	-	-	HPV4
24	CW260C	-	-	-	6.8×10^{-3}	-	**1.08×10^{5}**	-	-	-	-	HPV4
25	CW276	-	-	-	-	1.8×10^{-2}	-	-	-	-	-	WD

[a] Viral loads of causative HPV types are indicated in bold; WD, common wart without determined causative HPV type.
